# Factors Associated With Willingness to Become a Living Organ Donor

**DOI:** 10.1001/jamanetworkopen.2025.27592

**Published:** 2025-08-19

**Authors:** Taranika Sarkar Das, Allie Carter, Carolyn Marie Haugh Singleton, Lauren D. Nephew

**Affiliations:** 1 Department of Gastroenterology and Hepatology, The Brooklyn Hospital Center, New York, New York; 2 Department of Biostatistics and Health Data Science, Indiana University School of Medicine, Indianapolis; 3 Department of Medicine, Indiana University School of Medicine, Indianapolis; 4 Division of Gastroenterology and Hepatology, Department of Medicine, Indiana University School of Medicine, Indianapolis

## Abstract

**Importance:**

Organ transplant demand in the US far exceeds supply. Living donor organ transplantation (LDOT) offers a critical solution to reducing this shortage, yet its adoption remains limited. Understanding factors that influence willingness to donate is essential for expanding the living donor pool.

**Objective:**

To examine whether knowledge of LDOT, perceptions of fairness in the transplant system, geography, socioeconomic status, and religiosity are associated with individuals’ willingness to become living organ donors.

**Design, Setting, and Participants:**

This cross-sectional study analyzed responses from the 2019 National Survey of Organ Donation Attitudes and Practices (NSODAP). Participants were aged 18 years or older. Poststratification weights were applied to align the sample with US census demographic characteristics. Data were analyzed from November 2024 through May 2025.

**Exposures:**

Socioeconomic status, region of residence, knowledge of living donation, perceptions of inequality in transplantation, and religiosity.

**Main Outcome and Measures:**

The primary outcome was the self-reported likelihood of donating an organ to a family member while living. Composite scores were created to assess knowledge about living donation, perceptions of fairness in organ transplantation, and religiosity. Multivariable logistic regression was performed to evaluate associations with willingness to donate, adjusting for demographic and socioeconomic factors.

**Results:**

Of the 9922 survey respondents, 5264 (53.2%) were women, and 7801 (78.9%) reported an educational level of postsecondary training or above. The largest proportion of participants was aged 18 to 34 years (3006 [32.6%]), followed by 50 to 64 years (2390 [25.9%]), and most had health insurance (8547 [86.1%]); 8667 (87.4%) reported being likely to donate. Women were more likely to report willingness to donate than men (odds ratio [OR], 1.49; 95% CI, 1.23-1.81; *P* < .001), and Asian participants were less likely to report willingness to donate than White participants (OR, 0.72; 95% CI, 0.55-0.94; *P* = .02). Higher knowledge scores were associated with increased willingness to donate (OR, 1.62; 95% CI, 1.49-1.77; *P* < .001), while lower perceptions of fairness in organ allocation were associated with decreased willingness (OR, 0.85; 95% CI, 0.78-0.92; *P* < .001).

**Conclusions and Relevance:**

In this cross-sectional study analyzing survey data from NSODAP, willingness to become a living organ donor was associated with knowledge, perceptions of fairness within the organ transplant system, and socioeconomic factors. The findings suggest that targeted education and transparency in organ allocation policies may help expand the US living donor pool.

## Key Points

**Question:**

What factors are associated with US adults’ willingness to become a living organ donor to a family member?

**Findings:**

In this cross-sectional study of 9922 adults from the National Survey of Organ Donation Attitudes and Practices, greater knowledge of living donation, higher religiosity, and perceptions of fairness in the transplant system were associated with increased willingness to donate. Women and White participants were more likely to report willingness to donate, while Asian participants were less likely.

**Meaning:**

Efforts to expand the living donor pool may benefit from targeted education and strategies that promote trust and transparency in the transplant system.

## Teaser

This cross-sectional study uses data from a nationally representative survey to investigate factors associated with US adults’ willingness to be a living organ donor to a family member.

## Introduction

In the US, the demand for organs continues to far exceed the available supply.^1^ Living donor organ transplantation (LDOT) offers a critical strategy to reduce this gap by expanding the donor pool and lowering waiting list mortality. However, LDOT remains underused. For example, living donor liver transplantation accounts for only 15% to 20% of liver transplants in Western countries compared with over 90% in many Eastern countries.^2,3^ Similarly, living donor kidney transplantation represents roughly one-third of all kidney transplants in the US.^4^ In 2023, of 10 659 liver transplants performed in the US, only 555 (5.2%) involved living donors.^5^

Beyond overall underuse, persistent disparities in LDOT access have been documented. Racial and ethnic minority groups, particularly Black and Hispanic patients, who carry a disproportionately high burden of end-stage liver and kidney disease, are less likely to receive living donor transplants than White patients.^6^ Socioeconomic factors such as lower income, public insurance, and limited health care access further exacerbate these inequities.^7^

System-level and individual-level barriers both contribute to low LDOT uptake. In 2021, the American Society of Transplantation identified limited clinician awareness, inconsistent donor selection criteria, and insufficient long-term donor outcome data as key structural barriers.^7^ Patient-level obstacles such as financial insecurity, lack of insurance, and logistic challenges also play a substantial role in deterring living donation.^6^

Personal attitudes and trust in the health care system further influence donation decisions. According to a survey by the Health Resources and Services Administration (HRSA), nearly one-third of Americans reported they would not be willing to donate organs, even to a close relative, highlighting an underlying reluctance that may impact LDOT participation.^8^ Another US study found that while religious service attendance was not associated with willingness to be a deceased donor, trust in medical care was associated with donor registration.^9^ Additionally, highly publicized incidents, such as the 2023 case of a Kentucky man incorrectly declared brain dead prior to planned organ retrieval, have the potential to erode public trust in the transplant system.^10^

Knowledge gaps also hinder LDOT uptake. A single-center study found that most patients on the liver transplant waiting list were unaware that living liver donation was even an option. Among those who were aware, few understood its benefits, risks, or availability.^11^ These findings emphasize the need for standardized, accessible education strategies tailored to diverse patient populations.^12,13^

Improving LDOT adoption in the US requires a comprehensive understanding of the barriers and motivators that shape donation attitudes. This study used data from a nationally representative survey to evaluate factors associated with individuals’ willingness to become living organ donors. We focused on 5 key domains: knowledge of living donation, perceptions of fairness in the transplant system, socioeconomic status (SES), geography, and religiosity. By identifying modifiable attitudinal and structural drivers of willingness to donate, this work aimed to inform future efforts to expand the living donor pool and promote equitable access to transplantation.

## Methods

### Data Source

Data for this cross-sectional study were drawn from the 2019 National Survey of Organ Donation Attitudes and Practices (NSODAP), the fourth national survey assessing public views on organ donation and transplantation.^14^ The NSODAP was a cross-sectional national survey administered by the US Department of Health and Human Services, HRSA, and Health Systems Bureau Division of Transplantation. This was the fourth and most recent administration of the survey (previously conducted in 1993, 2005, and 2012). The full questionnaire is provided in Appendix E of the publicly available survey report,^15^ and the HRSA was the commissioning and funding body. To ensure demographic diversity, the survey used a multimode (telephone and online) split design, with telephone samples drawn from residential addresses using the Equal Probability of Selection Method and oversampling in zip codes with high concentrations of minority residents. The current study was reviewed by the Indiana University institutional review board, which determined the project was not human participants research and did not require approval or informed consent. The study adhered to the Strengthening the Reporting of Observational Studies in Epidemiology (STROBE) reporting guideline for cross-sectional studies.

Participants were aged 18 years or older and completed the survey in English or Spanish. Poststratification weights were applied to align the sample with US census demographic characteristics.

### Exposures and Covariates

Key exposures in this study were categorized into 5 domains to examine factors associated with organ donation attitudes. Socioeconomic status was measured using self-reported income, educational level, insurance type, and employment status. Geography was determined by US region of residence (Northeast, Midwest, South, or West) and rural vs urban residence. Knowledge of living donation was assessed through 4 questions that queried respondents about living donation possibilities for kidneys, liver segments, and lung parts and knowledge regarding the mortality risk for individuals on the transplant waiting list. Perceptions of inequality were gauged with 4 questions evaluating respondents’ beliefs about minority patients’ likelihood of receiving transplants, the fairness of the US transplant system, and perceived disparities in transplant access due to wealth. Religiosity was assessed by asking if respondents’ religion opposed organ donation and by measuring the importance of religious beliefs using a Likert scale. Knowledge, inequality and fairness, and religion were aggregated into 3 composite scores (knowledge: score range, 0-4, with higher scores indicating more knowledge; perceived inequality: score range, 0-4, with higher scores indicating lower perception of fairness; and religion: score range, 0-2, with higher scores indicating greater emphasis on religion), to avoid collinearity during modeling (eTable 1 in Supplement 1). Further details on the NSODAP questions used to define each exposure are provided in eTable 1 in Supplement 1.

Covariates included demographic information such as age, race, ethnicity, and gender, self-reported by survey participants. Race and ethnicity were included in the analysis because we hypothesized that that they may be associated with willingness to donate. Race was categorized using the NSODAP standardized categorizations: Asian, Black, Native American, White, and multiracial or other (not further specified in the survey). Ethnicity was categorized as Hispanic or Latino and not Hispanic or Latino. Gender was classified as men, women, and other.

### Outcome Measure

The primary outcome was the likelihood of living organ donation to a family member, assessed via the NSODAP question “How likely are you to donate an organ to a family member while living?” (eTable 1 in Supplement 1). Responses were dichotomized: “very likely” and “somewhat likely” were coded as *likely*, while “not very likely” and “not at all likely” were coded as *unlikely*.

### Statistical Analysis

Categorical variables were compared between groups (likely vs unlikely donors) using the Rao-Scott χ^2^ test. Continuous variables were assessed using the *t* test. Logistic regression was used to explore factors associated with the likelihood of donation. Initially, individual factors were analyzed separately in univariable models to assess their association with the likelihood of organ donation. We identified that certain factors within the domains of knowledge, fairness, and religion not only were associated with donation likelihood but also had significant correlations with other variables. To mitigate issues of multicollinearity—where factors may overlap and obscure their individual outcomes—as well as to aide in clinical interpretability, composite scores were created for each domain. Internal consistency and reliability were examined within the domains using correlation matrices and Cronbach α. These composite scores, along with demographic and socioeconomic characteristics associated with the outcome, were then incorporated into a multivariable model to provide a more accurate analysis of factors associated with donation likelihood. Weighting was applied to ensure the NSODAP sample reflected US census characteristics. Collinearity among demographic and socioeconomic covariates was assessed using variance inflation factors, all of which were below the acceptable threshold of 5, confirming the absence of multicollinearity.

As a sensitivity analysis, an SES summation score ranging from 0 to 5, with higher scores indicating more indicators of adverse SES, was also created, where 1 point was given for each of the following: annual income less than $20 000, educational level of high school graduate or below, not having private insurance, unemployed or retired, and not married or partnered. Multivariable models were run with each SES variable individually and then with the SES summation score. All analyses were conducted from November 2024 through May 2025 using SAS, version 9.4 (SAS Institute Inc), and R, version 4.1.0 (R Project for Statistical Computing). Statistical significance was set at *P* < .05, and nonparametric tests were used where appropriate.

## Results

The 2019 NSODAP survey included 10 000 respondents, with 2000 completing the survey by telephone and 8000 online. Despite the option to skip questions, the survey achieved a high completion rate, with telephone respondents answering approximately 95% of the questions and web respondents, approximately 99%, as reported in the NSODAP. The full sample of the current study included 9922 adults who completed the outcome question, reflecting the demographic and geographic diversity of the national population. A total of 4571 respondents (46.2%) were men, 5264 (53.2%) were women, and 60 (0.6%) were other gender. By race category, 1040 respondents (10.5%) identified as Asian, 1048 (10.6%) as Black, 795 (8.0%) as Native American, 6661 (67.1%) as White, and 378 (3.8%) as other race or multiracial. A total of 1095 respondents (11.0%) were Hispanic or Latino and 8818 (89.0%) were not Hispanic or Latino. The largest proportion of respondents was aged 18 to 34 years (3006 [32.6%]), followed by 50 to 64 years (2390 [25.9%]), and over half (7801 [78.9%]) had an educational level of postsecondary training or above. Most respondents reported having health insurance (8547 [86.1%]).

### Demographic Differences in Likelihood to Donate

**Figure 1.**
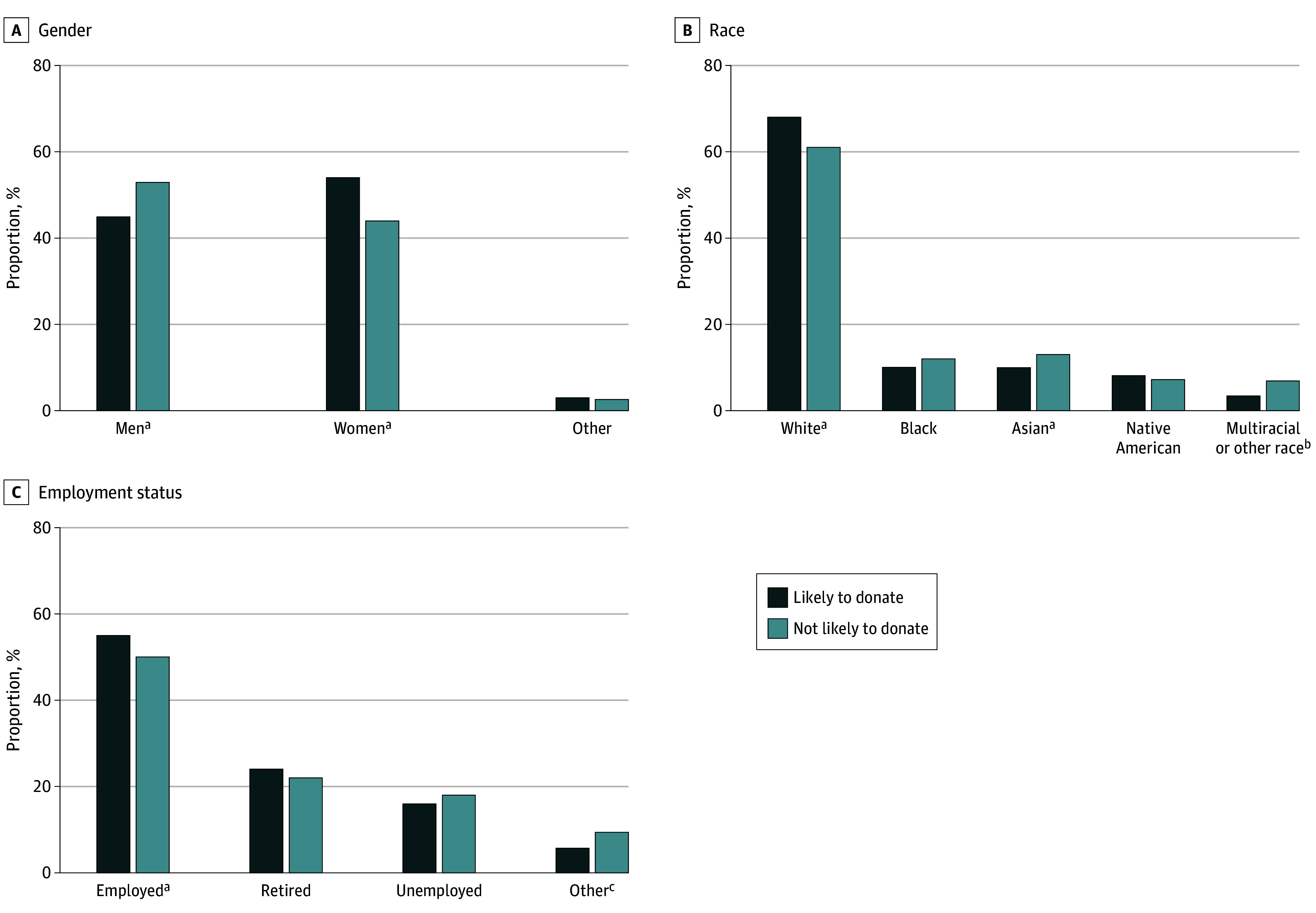
Comparison of the Likelihood to Be vs Not to Be a Living Organ Donor Among Participants by Race, Gender, and Employment Status ^a^Associated with the likelihood of living organ donation on multivariable analysis. ^b^Other race was not further specified in the survey. ^c^Other employment status includes taking care of home or family but not working, student, unable to work, or other.

Of the 9922 participants who completed the outcome question, 8667 (87.4%) were classified as likely to donate (LD) and 1255 (12.6%) as not likely to donate (NL) (Table 1). Age distribution did not differ between the 2 groups. However, there were notable racial differences: White participants were more represented in the LD group (5897 [68.0%]) than the NL group (764 [60.9%]), while Black participants were more represented in the NL group (146 [11.6%]) than LD (902 [10.4%]) (*P* < .001). Participants who were Asian or identified as multiracial or other race also had higher representation in the NL than LD group (Asian, 168 [13.4%] NL vs 872 [10.1%] LD; multiracial or other race, 87 [6.9%] NL vs 291 [3.4%] LD) (Table 1 and Figure 1).

**Table 1.** Demographics and Socioeconomic Characteristics Among Participants Likely and Not Likely to Be a Living Organ Donor

Characteristic	Participants, No. (%) (N = 9922)^a^		*P* value
	Likely to be a donor (n = 8667)	Not likely to be a donor (n = 1255)	
Age, y			
18-34	2663 (32.6)	343 (32.7)	.79
35-49	1692 (20.7)	212 (20.2)	
50-64	2107 (25.8)	283 (27.0)	
≥65	1707 (20.9)	210 (20.0)	
Missing	498	207	
Race			
Asian	872 (10.1)	168 (13.4)	<.001
Black	902 (10.4)	146 (11.6)	
Native American	705 (8.1)	90 (7.2)	
White	5897 (68.0)	764 (60.9)	
Other or multiracial^b^	291 (3.4)	87 (6.9)	
Ethnicity			
Hispanic or Latino	937 (10.8)	158 (12.6)	.18
Not Hispanic or Latino	7722 (89.2)	1096 (87.4)	
Missing	8	1	
Gender			
Men	3907 (45.2)	664 (53.2)	<.001
Women	4713 (54.5)	551 (44.2)	
Other	28 (0.3)	32 (2.6)	
Missing	19	8	
Annual income, $			
<20 000	1073 (14.6)	264 (24.3)	<.001
20 000-40 000	1753 (23.8)	250 (23.0)	
50 000-100 000	2465 (33.5)	321 (29.5)	
>100 000	2069 (28.1)	252 (23.2)	
Missing	1307	168	
Educational level			
High school graduate or below	1767 (20.5)	316 (25.3)	<.001
Postsecondary training or above	6866 (79.5)	935 (74.7)	
Missing	34	4	
Works in health care			
Yes	991 (11.5)	109 (8.7)	.050
No	7643 (88.5)	1143 (91.3)	
Missing	33	3	
Insurance			
Government or Medicaid	3104 (40.8)	455 (48.2)	<.001
Medicare	138 (1.8)	20 (2.1)	
Private	3954 (52.0)	433 (45.9)	
VA or TRICARE	390 (5.1)	35 (3.7)	
Other	17 (0.2)	1 (0.1)	
Missing	1064	311	
Marital status			
Married or partnered	3801 (44.2)	672 (54.1)	<.001
Not married or partnered	4790 (55.8)	570 (45.9)	
Missing	76	13	
Employment status			
Employed part or full time	4733 (55.0)	625 (50.0)	<.001
Retired	2051 (23.8)	279 (22.3)	
Unemployed	1337 (15.5)	230 (18.4)	
Other^c^	487 (5.7)	117 (9.4)	
Missing	59	4	
US region			
Northeast	1405 (16.3)	249 (20.1)	.01
Midwest	1815 (21.1)	231 (18.6)	
South	2921 (33.9)	388 (31.3)	
West	2470 (28.7)	371 (29.9)	
Missing	56	16	
Rurality			
Rural	1322 (16.3)	165 (14.1)	.15
Urban	6777 (83.7)	1006 (85.9)	
Missing	568	84	
Religion category			
Atheist or agnostic	860 (12.7)	116 (14.2)	.04
Christian	4267 (62.9)	487 (59.8)	
Jewish	214 (3.2)	34 (4.2)	
Muslim	90 (1.3)	17 (2.1)	
Other religion	1350 (19.9)	161 (19.8)	
Missing	1886	440	

Abbreviation: VA, Veterans Affairs.

^a^ Poststratification weights were applied to align the sample with US census demographic characteristics.

^b^ Other race was not further specified in the survey.

^c^ Other employment included taking care of home or family but not working, student, unable to work, or other.

Most participants in the LD group were women (4713 [54.5%], vs 551 [44.2%] in the NL group), whereas most of those not likely to donate were men (664 [53.2%] NL vs 3907 [45.2%] LD). More respondents identified as other gender in the NL group (32 [2.6%]) than in the LD group (28 [0.3%]). Ethnicity was not associated with likelihood to be a living donor, with Hispanic or Latino respondents accounting for 937 participants (10.8%) in the LD group and 158 (12.6%) in the NL group (*P* = .18) (Table 1, Figure 1).

### Geography

Geographic differences were apparent between groups. Individuals from the Northeast (249 [20.1%] NL vs 1405 [16.3%] LD) and West (371 [29.9%] NL vs 2470 [28.7%] LD) were more represented in the NL group, while participants from the Midwest (1815 [21.1%] LD vs 231 [18.6%] NL) and South (2921 [33.9%] LD vs 388 [31.3%] NL) were more represented in the LD group (*P* = .01) (Table 1).

### Socioeconomic Factors

Only 1073 of the participants likely to donate (14.6%) reported annual income below $20 000, compared with 264 of those not likely to donate (24.3%) (*P* < .001). Participants with a high school education or below were more represented in the NL (316 [25.3%]) than the LD (1767 [20.5%]) group, whereas those with postsecondary training or above were more represented in the LD (6866 [79.5%]) than NL (935 [74.7%]) group (*P* < .001). Employment status was also associated with donation likelihood; employed individuals were more represented in the LD (4733 [55.0%]) than NL (625 [50.0%]) group, while unemployed participants were more represented in the NL (230 [18.4%]) than LD (1337 [15.5%]) group (*P* < .001) (Table 1, Figure 1).

Insurance type was another factor significantly associated with likelihood of being a living donor. Participants with government insurance or Medicaid were more represented in the NL (455 [48.2%]) vs LD (3104 [40.8%]) group, while those privately insured or with Veterans Affairs (VA) or TRICARE insurance were more represented in the LD group (private insurance: 3954 [52.0%] LD vs 433 [45.9%] NL; VA or TRICARE: 390 [5.1%] LD vs 35 [3.7%] NL) (*P* < .001). Additionally, individuals working in health care were more represented in the LD (991 [11.5%]) vs NL (109 [8.7%]) group, while those not working in health care were more represented in the NL (1143 [91.3%]) vs LD (7643 [88.5%]) group (*P* = .05) (Table 1).

### Knowledge of Living Donation

Knowledge about living donation was higher in the LD group. For instance, 8044 participants (93.8%) in the LD group knew that kidneys could be donated by a living donor, compared with 876 (70.5%) of those in the NL group (*P* < .001). Similarly, 6933 (82.2%) in the LD group were aware that parts of livers could be donated by a living donor, compared with 712 (58.3%) in the NL group (*P* < .001), and awareness of lung donation also followed this pattern (4906 [60.3%] in the LD vs 544 [45.0%] in the NL group knew that parts of lungs could be donated by a living donor; *P* < .001). The mean (SD) knowledge score was also higher for the LD (3.20 [0.92]) than NL (2.36 [1.43]) group (*P* < .001) (Table 2).

**Table 2.** Association of Knowledge, Perceived Inequality in Transplantation, and Religiosity With the Likelihood to Be a Living Organ Donor

Characteristic	Participants^a^		*P* value
	Likely to be a donor (n = 8667)	Not likely to be a donor (n = 1255)	
**Knowledge about transplantation**			
Kidneys can be donated from a living donor			
Yes	8044 (93.8)	876 (70.5)	<.001
No	534 (6.2)	367 (29.5)	
Missing	89	12	
Parts of livers can be donated from a living donor			
Yes	6933 (82.2)	712 (58.3)	<.001
No	1497 (17.8)	509 (41.7)	
Missing	237	34	
Parts of lungs can be donated from a living donor			
Yes	4906 (60.3)	544 (45.0)	<.001
No	3225 (39.7)	665 (55.0)	
Missing	536	46	
Many people on the national transplant waiting list die because the organ they need is not donated in time			
Agree	7836 (92.3)	822 (66.6)	<.001
Disagree	658 (7.7)	412 (33.4)	
Missing	173	21	
Knowledge score^b^			
Mean (SD) [range]	3.20 (0.92) [0.00-4.00]	2.36 (1.43) [0.00-4.00]	<.001
Missing	9	2	
**Inequality and fairness**			
Minority patients are less likely to receive organ transplants			
Agree	4311 (52.0)	510 (42.5)	<.001
Disagree	3985 (48.0)	689 (57.5)	
Missing	371	56	
The US transplant system uses a fair approach to distribute organs to patients			
Agree	6056 (74.7)	582 (49.0)	<.001
Disagree	2047 (25.3)	605 (51.0)	
Missing	564	68	
Given equal need, a poor person has as good a chance as a rich person of getting an organ transplant			
Agree	4511 (53.1)	524 (42.4)	<.001
Disagree	3984 (46.9)	711 (57.6)	
Missing	172	20	
Transplants often go to undeserving people			
Agree	2589 (31.1)	401 (33.1)	0.81
Disagree	5747 (68.9)	810 (66.9)	
Missing	331	44	
Inequality score^c^			
Mean (SD) [range]	1.50 (1.10) [0.00-4.00]	1.78 (1.05) [0.00-4.00]	<.001
Missing	44	4	
**Religiosity**			
Organ donation is against my religion			
Agree	1373 (16.0)	325 (26.2)	<.001
Disagree	7192 (84.0)	916 (73.8)	
Missing	102	14	
How important are your religious beliefs?			
Very important	3781 (44.1)	404 (32.7)	<.001
Somewhat important	2541 (29.6)	337 (27.2)	
Not very important	1041 (12.1)	207 (16.7)	
Not at all important	1210 (14.1)	289 (23.4)	
Missing	94	18	
Religion score^d^			
Mean (SD) [range]	1.51 (0.49) [0.00-2.00]	1.29 (0.53) [0.00-2.00]	<.001
Missing	24	3	

^a^ Data are presented as number (percentage) of participants unless otherwise noted. Poststratification weights were applied to align the sample with US census demographic characteristics.

^b^ Knowledge score ranges from 0 to 4, with higher scores indicating more knowledge.

^c^ Inequality score ranges from 0 to 4, with higher scores indicating lower perception of fairness.

^d^ Religion score ranges from 0 to 2, with higher scores indicating greater emphasis on religion.

### Perceptions of Inequality and Fairness

Perceptions of fairness in the transplant system varied between groups. Among those in the LD group, 4311 (52.0%) believed that minority patients were less likely to receive organ transplants, compared with 510 (42.5%) in the NL group (*P* < .001). Additionally, 6056 (74.7%) in the LD group felt that the US transplant system fairly distributes organs, compared with only 582 (49.0%) in the NL group (*P* < .001). The mean (SD) inequality score was lower among those in the LD group (1.50 [1.10]) vs the NL group (1.78 [1.05]) (*P* < .001) (Table 2).

### Religiosity

Religiosity also influenced donation likelihood, with higher religiosity scores associated with a greater likelihood of donation. Among those in the LD group, 3781 (44.1%) reported that religious beliefs were “very important” to them, compared with 404 (32.7%) of those in the NL group (*P* < .001). Additionally, 7192 (84.0%) in the LD group disagreed with the statement that their religion opposes organ donation, compared with 916 (73.8%) in the NL group (*P* < .001) (Table 2).

### Factors Associated With Likelihood of Living Donor Organ Donation

**Figure 2.**
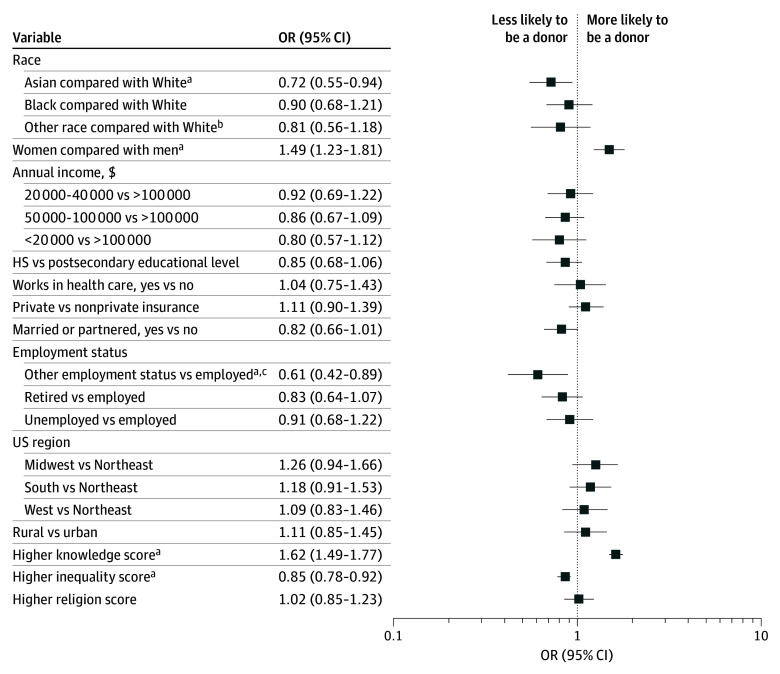
Multivariable Analysis of Factors Associated With Likelihood of Being a Living Organ Donor HS indicates high school; and OR, odds ratio. Whiskers represent 95% CIs. ^a^*P* < .05. ^b^Other race was not further specified in the survey. ^c^Other employment status includes taking care of home or family but not working, student, unable to work, or other.

In univariable analysis, many factors were associated with donation, including race, income, gender, insurance type, employment, region, knowledge score, inequality score, and religiosity score (eTable 2 in Supplement 1). In multivariable analysis, women remained more likely to be in the LD group than men (odds ratio [OR], 1.49; 95% CI, 1.23-1.81; *P* < .001), and Asian participants were less likely to be in the LD group than White participants (OR, 0.72; 95% CI, 0.55-0.94; *P* = .02) (Figure 2). Other employment types listed on the survey (taking care of home or family but not working, student, unable to work, or other), compared with being employed, were associated with lower odds of being in the LD group (OR, 0.61; 95% CI, 0.42-0.89; *P* = .01). Higher knowledge scores were associated with being in the LD group (OR, 1.62; 95% CI, 1.49-1.77; *P* < .001). Additionally, lower perceptions of fairness in the transplant system (higher inequality scores) were associated with lower odds of being in the LD group (OR, 0.85; 95% CI, 0.78-0.92; *P* < .001). Being in the LD group was not associated with income ($20 000-$40 000 vs >$100 000: OR, 0.92; 95% CI, 0.69-1.22; *P* = .55; $50 000-$100 000 vs >$100 000: OR, 0.86; 95% CI, 0.67-1.09; *P* = .20; <$20 000 vs >$100 000: OR, 0.80; 95% CI, 0.57-1.12; *P* = .20), educational level (high school graduate or below vs postsecondary training or above: OR, 0.85; 95% CI, 0.68-1.06; *P* = .15), or insurance type (private vs nonprivate: OR, 1.11; 95% CI, 0.90-1.39; *P* = .33) in multivariable analysis (Figure 2).

**Figure 3.**
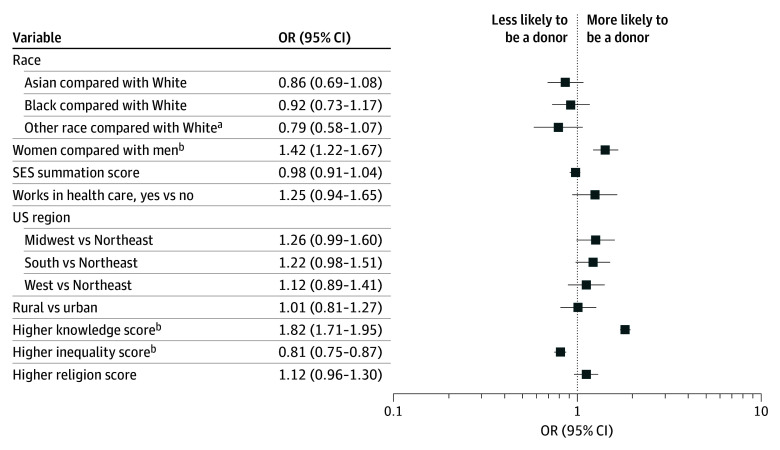
Multivariable Model for Socioeconomic Status (SES) Summation Score Sensitivity OR indicates odds ratio. Whiskers represent 95% CIs. ^a^Other race was not further specified in the survey. ^b^*P* < .05.

In a sensitivity analysis, the SES summation score was added to the multivariable model and was not associated with being in the LD group (OR, 0.98; 95% CI, 0.91-1.04; *P* = .44) (Figure 3). Female gender, knowledge score, and inequality score remained significantly associated with likelihood of being a living donor (Figure 3).

## Discussion

Our study examined the association of knowledge, perceptions of fairness in the transplant system, and religiosity with individuals’ likelihood to consider LDOT. Findings revealed that increased religiosity, knowledge about LDOT, and a perception of fairness in the system were all associated with a higher likelihood of donation. Of note, gender, geography, race, income, and educational attainment emerged as significant demographic factors associated with willingness to donate, underscoring the importance of understanding how these elements impact donor likelihood in diverse populations.

In this study’s sample, religiosity was positively associated with the likelihood to donate. This finding challenges the assumption that religious beliefs may act as a barrier to LDOT and aligns with previous studies showing that while certain religious beliefs can influence health decisions, many individuals regard donation as an altruistic act compatible with their beliefs.^16^ Additionally, female gender and White race were associated with a higher likelihood of donation. This finding is consistent with prior research investigating patterns across donor demographics and suggests that societal expectations and socioeconomic factors influence donation tendencies.^17^ Similar trends have been observed in smaller, population-specific studies. For instance, a survey of emergency department patients found that White individuals and women were more likely to report willingness to register as organ donors,^18^ and studies of health sciences students have similarly reported higher willingness to donate among female participants and those with greater knowledge of transplantation.^19^

Knowledge emerged as a critical factor, as participants with a higher awareness of living organ donation options were more likely to consider donating. Prior studies have highlighted the role of transplant centers in improving patient knowledge, pointing to factors like community engagement, awareness of culturally specific barriers, and quality of information provided as pivotal in addressing disparities.^20^ However, to our knowledge there are still no standardized recommendations for educating patients or the public specifically about living donation practices and outcomes. A review of content provided by transplant centers about living donation across 11 regions revealed notable inconsistencies,^12^ and a review of living donation information available on Twitter (now X) showed similar variation in the information shared.^13^ In our study, increased knowledge scores among likely donors underscore the importance of effective, accessible education efforts that can bridge information gaps, particularly for minority communities. More efforts must be made to increase public access to reliable information about living donation. Mandating standardized education about living donation for all patients undergoing transplant evaluation, even those without an identified potential donor, may offer a systematic and scalable pathway to disseminate accurate information and counter misinformation within communities.

The perception of fairness in the transplant system also was associated with the donation likelihood. Participants who believed that the system fairly distributed organs or who felt confident in equitable access were more likely to consider donation. While medical mistrust is often shaped by individual experiences across the broader health system, our findings suggest a specific, nationally observed pattern of distrust toward the transplant field. This finding suggests that perceptions of biases and mistrust within the transplant system may deter potential donors, particularly among racial and ethnic minority populations. A recent review of interventions to increase organ donation showed that providing education through peer leaders could be promising in bolstering donation rates.^21^ Additionally, culturally sensitive community outreach could improve organ availability and reduce disparities, as evidenced by the success of a campaign targeted to increase donation rates in a Hispanic population in Southern California.^22^

### Implications for Improving the Status Quo

Our study findings support the increasing call to reduce inequity present in the current transplant system, improve public trust in transplantation, and expand awareness about LDOT. There have been multiple novel interventions trialed at living donor kidney transplantation centers across the country to bolster LD, including home-based and telehealth programs,^23,24^ patient navigators,^25^ culturally competent care programs,^26^ and financial assistance provided to donors.^27^ These programs have all demonstrated small-scale efficacy in increasing LDOT in the kidney transplant realm on the patient level and provide evidence that trust-building and education improve uptake. However, scaling individual interventions may fall short of meeting national needs, particularly for expanding access to living liver or lung donations, which present additional complexities. Current evidence suggests that culturally competent care models and peer-led or social network–based engagement strategies are more effective than financial or policy-based incentives in promoting living donation.^26,28,29,30^ A strategic-development task force, potentially led by the United Network for Organ Sharing, could play a central role in expanding evidence-based interventions, targeting underengaged populations and geographic regions identified in our study and other studies^31^ and ultimately advancing national efforts to strengthen living donor programs.

National public health interventions aimed at improving transparency and reducing inequities are likely to have broader and more sustained impact in addressing the public concerns identified in our study. However, prior reviews of state-level donation policies, such as donor registry mandates, tax incentive programs, and specialty license plate campaigns, have found limited evidence of effectiveness in increasing actual donation rates.^32^ One notable exception has been policies that generate revenue pools, or dedicated funding streams—often from department of motor vehicles opt-in donations or check-off programs—used to support donation-related outreach and infrastructure. Strengthening existing national frameworks, such as those led by the United Network for Organ Sharing and HRSA, through dedicated revenue pools could help fund tailored community education campaigns to promote living donation, offering a more flexible and sustainable alternative to prior legislative strategies.

Currently, there is a paucity of standardized and patient-centered data available to target inequity and to aid potential donors in making informed decisions.^33,34^ Promoting publicly available resources such as the Living Donor Collective, a national registry led by the Scientific Registry of Transplant Recipients to longitudinally track donor outcomes,^35^ and the Living Donation Storytelling Project, an online platform where diverse donors and recipients share personal experiences through video narratives, could enhance access to transparent, patient-friendly education.^36^ Additionally, multiple publications have called for standardizing how organ procurement organizations (OPOs) report performance data^37,38^ and for accessible, nonpunitive feedback to target inequities.^39^ Leveraging accessible and transparent feedback on OPO and transplant center outcomes, combined with equity-focused quality improvement initiatives, has the potential to concretely identify and reduce disparities and, thus, improve public trust in transplantation.^40^

There may be a concern that calling for national reform and transparency could further sour public opinion. However, during the Centers for Medicare & Medicaid Services public debate on OPO performance in 2019-2020, national donation rates increased.^41^ This trend suggests that national legislative reforms aimed at improving transparency in organ donation may strengthen trust rather than undermine it. Alongside legislative reform and clearer metrics, unbiased, culturally tailored, and factual public health campaigns about LDOT are needed on the national scale to improve public awareness and knowledge about transplantation.^42,43^

### Limitations

This study has limitations. It primarily reflects the general public’s attitudes toward living donation rather than the views of individuals actively considering or having completed organ donation. The use of self-reported data and a multimode survey design may also introduce response biases. Although weighting was applied to align the sample with US demographics, residual biases may exist, particularly among underrepresented subgroups. Additionally, while some differences may appear modest, their significance is amplified at the population level. Given that this study was designed to be nationally representative, these findings likely reflect real differences in public opinion that, if addressed through targeted interventions, could lead to meaningful impact on a broad scale. In addition, while the primary outcome was dichotomized to reflect directional intent, this approach may obscure nuances among respondents who were ambivalent (eg, “somewhat likely”), a group that may be especially responsive to targeted education or trust-building interventions.

## Conclusions

In this analysis of cross-sectional survey data from the NSODAP, willingness to become a living organ donor was associated with knowledge, perceptions of fairness within the organ transplant system, and socioeconomic factors. These findings underscore the need for multifaceted interventions that address barriers and motivators, aiming to expand the living donor pool and promote equity in transplantation. Future work should continue to explore how transplant centers and organ procurement organizations can leverage these insights to foster a more inclusive approach to living organ donation.

## Notes

Supplement 1. **eTable 1.** NSODAP Questions Defining Exposures and Outcomes **eTable 2. **Univariable Logistic Regression

Supplement 2.
Data Sharing Statement
