# Supplementary material for: Factors Associated With Willingness to Become a Living Organ Donor
Source: JAMA Netw Open. 2025 Aug 19;8(8):e2527592. doi: 10.1001/jamanetworkopen.2025.27592 (PMC12365702; doi:10.1001/jamanetworkopen.2025.27592)
Supplement: Supplement 1. — eTable 1. NSODAP Questions Defining Exposures and Outcomes eTable 2. Univariable Logistic Regression [file jamanetwopen-e2527592-s001.pdf]

Supplementary Online Content

Sarkar Das T, Carter A, Singleton CMH, Nephew LD. Factors associated with willingness to become a living organ donor. *JAMA Netw Open*. 2025;8(8):e2527592. doi:10.1001/jamanetworkopen.2025.27592

eTable 1. NSODAP Questions Defining Exposures and Outcomes

eTable 2. Univariable Logistic Regression

This supplementary material has been provided by the authors to give readers additional information about their work.

© 2025 Sarkar Das TS et al. *JAMA Network Open*.

eTable 1: NSODAP Questions Defining Exposures and Outcomes

Variable	NSODAP Question Numbers	Question Description
**Knowledge of Living Donation**	Q110, Q111, Q112, Q113	Q110: Can kidneys be donated from a living donor? (Yes/No)
		Q111: Can parts of livers be donated from a living donor? (Yes/No)
		Q112: Can parts of lungs be donated from a living donor? (Yes/No)
		Q113: Many people on the transplant waiting list die due to lack of donated organs (Agree/Disagree)
**Perceptions of Inequality and Fairness**	Q115, Q116, Q117, Q118	Q115: Minority patients are less likely to receive organ transplants (Agree/Disagree)
		Q116: The U.S. transplant system uses a fair approach to distribute organs (Agree/Disagree)
		Q117: A poor person has the same chance as a rich person of getting an organ transplant (Agree/Disagree)
		Q118: Transplants often go to undeserving people (Agree/Disagree)
**Religiosity**	Q129, Q130	Q129: Organ donation is against my religion (Agree/Disagree)
		Q130: How important are your religious beliefs? (4-point Likert scale)
**Outcome (Likelihood to Donate)**	Q118	How likely are you to donate an organ to a family member while living? (Very likely, Somewhat likely, Not likely)

© 2025 Sarkar Das TS et al. *JAMA Network Open*.

eTable 2. Univariable Logistic Regression

Characteristic	Odds Ratio Estimates	95% Confidence Intervals		p-value
Demographics and SES				
**Age Category**	--	--	--	0.77
Age Category (35 - 49 vs 18 - 34)	1.05	0.84	1.31	0.66
Age Category (50 - 64 vs 18 - 34)	0.99	0.81	1.21	0.92
Age Category (65plus vs 18 - 34)	1.10	0.89	1.37	0.38
**Race Category**	--	--	--	<.001
Race Category (Asian vs White)	0.68	0.56	0.83	<.001
Race Category (Black vs White)	0.79	0.64	0.97	0.03
Race Category (Other[1] vs White)	0.54	0.42	0.70	<.001
**Ethnicity (Hispanic vs. Non-Hispanic)**	0.87	0.70	1.07	0.18
**Sex (Female vs Male)**	1.53	1.32	1.76	<.001
**Income**	--	--	--	<.001
Income ($20K-$40K vs >$100K)	0.86	0.69	1.07	0.17
Income ($50K-$100K vs >$100K)	0.90	0.73	1.11	0.33
Income (<$20K vs >$100K)	0.51	0.41	0.64	<.001
**Education (High School Graduate or Below vs. Post Secondary or Above)**	0.75	0.65	0.87	<.001
**Works in Healthcare (Yes vs. No)**	1.28	1.00	1.64	0.050
**Insurance (Private vs. Non-Private)**	1.36	1.15	1.60	<.001
**Married vs. Not Married**	0.64	0.56	0.74	<.001
**Employment**	--	--	--	<.001
Employment (Other vs Employed)	0.51	0.39	0.65	<.001
Employment (Retired vs Employed)	1.00	0.83	1.19	0.95
Employment (Unemployed vs Employed)	0.77	0.63	0.94	0.01
Geographic Characteristics				
**Region**	--	--	--	0.01
Region (Midwest vs Northeast)	1.42	1.13	1.79	0.003
Region (South vs Northeast)	1.32	1.08	1.62	0.007
Region (West vs Northeast)	1.22	0.99	1.51	0.06
**Rural vs. Urban**	1.17	0.95	1.43	0.15
Knowledge about Transplantation				
**Kidneys can be donated from a living donor. (Yes vs No)**	5.85	4.89	7.01	<.001
**Parts of livers can be donated from a living donor. (Yes vs No)**	3.08	2.64	3.58	<.001
**Parts of lungs can be donated from a living donor. (Yes vs No)**	1.81	1.56	2.09	<.001
**Many people on the national transplant waiting list die because the organ they need isn't donated in time. (Agree vs. Disagree)**	6.07	5.12	7.21	<.001
Inequality and Fairness				

© 2025 Sarkar Das TS et al. *JAMA Network Open*.

Characteristic	Odds Ratio Estimates	95% Confidence Intervals		p-value
**Minority patients are less likely to receive organ transplants. (Agree vs. Disagree)**	1.46	1.26	1.69	<.001
**The U.S. transplant system uses a fair approach to distribute organs to patients. (Agree vs. Disagree)**	3.19	2.75	3.71	<.001
**Given equal need, a poor person has as good a chance as a rich person of getting an organ transplant. (Agree vs. Disagree)**	1.62	1.41	1.88	<.001
**Transplants often go to undeserving people. (Agree vs. Disagree)**	1.02	0.87	1.19	0.81
Religion				
**Organ donation is against my religion. (Agree vs. Disagree)**	0.63	0.53	0.74	<.001
**How important are your religious beliefs?**	--	--	--	<.001
How important are your religious beliefs? (Not Very Important vs Not at All Important)	1.38	1.09	1.75	0.007
How important are your religious beliefs? (Somewhat Important vs Not at All Important)	2.09	1.70	2.57	<.001
How important are your religious beliefs? (Very Important vs Not at All Important)	2.66	2.19	3.25	<.001
Composite Scores				
**Knowledge Score**	1.90	1.78	2.02	<.001
**Inequality and Fairness Score**	0.79	0.74	0.84	<.001
**Religion Score**	1.20	1.04	1.39	0.02

[1]Other category contains race categories: Native American, Multiple/Other

© 2025 Sarkar Das TS et al. *JAMA Network Open*.
