# Supplementary material for: Factors Associated With Willingness to Become a Living Organ Donor
Source: JAMA Netw Open. 2025 Aug 19;8(8):e2527592. doi: 10.1001/jamanetworkopen.2025.27592 (PMC12365702; doi:10.1001/jamanetworkopen.2025.27592)
Supplement: Supplement 2. — Data Sharing Statement [file jamanetwopen-e2527592-s002.pdf]

Data Sharing Statement

Sarkar Das. Factors Associated With Willingness to Become a Living Organ Donor. *JAMA Netw Open*. Published August 19, 2025. doi:10.1001/jamanetworkopen.2025.27592

Data
Data available: Yes
Data types: Deidentified participant data
How to access data: All data relevant to the study are included in the article, uploaded as online supplemental information, or publicly available.
When available: With publication

Supporting Documents
Document types: Other (please specify)
Additional Information: All data relevant to the study are included in the article, uploaded as online supplemental information, or publicly available.NSODAP was a cross-sectional national survey administered by the U.S. Department of Health and Human Services (HHS), Health Resources and Services Administration (HRSA), Healthcare Systems Bureau, Division of Transplantation. This was the fourth and most recent administration of the survey (previously conducted in 1993, 2005, and 2012. The full questionnaire is provided in Appendix E of the publicly available survey report, and HRSA was the commissioning and funding body under federal contract GS10F0443Y/HHSH250201700044G.
How to access documents: All data relevant to the study are included in the article, uploaded as online supplemental information, or publicly available.NSODAP was a cross-sectional national survey administered by the U.S. Department of Health and Human Services (HHS), Health Resources and Services Administration (HRSA), Healthcare Systems Bureau, Division of Transplantation. This was the fourth and most recent administration of the survey (previously conducted in 1993, 2005, and 2012. The full questionnaire is provided in Appendix E of the publicly available survey report, and HRSA was the commissioning and funding body under federal contract GS10F0443Y/HHSH250201700044G.
When available: With publication

Additional Information
Who can access the data: All data relevant to the study are included in the article, uploaded as online supplemental information, or publicly available.NSODAP was a cross-sectional national survey administered by the U.S. Department of Health and Human Services (HHS), Health Resources and Services Administration (HRSA), Healthcare Systems Bureau, Division of Transplantation. This was the fourth and most recent administration of the survey (previously conducted in 1993, 2005, and 2012. The full questionnaire is provided in Appendix E of the publicly available survey report, and HRSA was the commissioning and funding body under federal contract GS10F0443Y/HHSH250201700044G.
Types of analyses: All data relevant to the study are included in the article, uploaded as online supplemental information, or publicly available.NSODAP was a cross-sectional national survey administered by the U.S. Department of Health and Human Services (HHS), Health Resources and Services Administration (HRSA), Healthcare Systems Bureau, Division of Transplantation. This was the fourth and most recent administration of the survey (previously conducted in 1993, 2005, and 2012. The full questionnaire is provided in Appendix E of the publicly available survey report, and HRSA was the commissioning and funding body under federal contract GS10F0443Y/HHSH250201700044G.
Mechanisms of data availability: All data relevant to the study are included in the article, uploaded as online supplemental information, or publicly available.NSODAP was a cross-sectional national survey administered by the U.S. Department of Health and Human Services (HHS), Health Resources and Services Administration (HRSA), Healthcare Systems Bureau,

Division of Transplantation. This was the fourth and most recent administration of the survey (previously conducted in 1993, 2005, and 2012. The full questionnaire is provided in Appendix E of the publicly available survey report, and HRSA was the commissioning and funding body under federal contract GS10F0443Y/HHSH250201700044G.
